# Scrounging by foragers can resolve the paradox of enrichment

**DOI:** 10.1098/rsos.160830

**Published:** 2017-03-01

**Authors:** Wataru Toyokawa

**Affiliations:** 1 School of Biology, University of St Andrews, St Andrews, Fife KY16 9TH, UK; 2 Japan Society for the Promotion of Science, Kojimachi, Chiyoda-ku, Tokyo 102-0083, Japan

**Keywords:** paradox of enrichment, community stability, predator–prey, producer–scrounger game, kleptoparasitism, social foraging

## Abstract

Theoretical models of predator–prey systems predict that sufficient enrichment of prey can generate large amplitude limit cycles, paradoxically causing a high risk of extinction (the paradox of enrichment). Although real ecological communities contain many gregarious species, whose foraging behaviour should be influenced by socially transmitted information, few theoretical studies have examined the possibility that social foraging might resolve this paradox. I considered a predator population in which individuals play the producer–scrounger foraging game in one-prey-one-predator and two-prey-one-predator systems. I analysed the stability of a coexisting equilibrium point in the one-prey system and that of non-equilibrium dynamics in the two-prey system. The results revealed that social foraging could stabilize both systems, and thereby resolve the paradox of enrichment when scrounging behaviour (i.e. kleptoparasitism) is prevalent in predators. This suggests a previously neglected mechanism underlying a powerful effect of group-living animals on the sustainability of ecological communities.

## 1. Introduction

Understanding how complex biological communities can persist has been an essential theme in ecology. For decades, community ecologists have attempted to reveal mechanisms that result in the maintenance or demise of natural communities [1–3]. One of the most intriguing predictions from classical predator–prey models is that sufficient enrichment of prey (i.e. increasing prey carrying capacity) can destabilize a natural community, causing a high risk of extinction [2]. This is called the paradox of enrichment. Although this hypothesis has been supported in simple predator–prey systems [4–6], it has been rejected by a number of empirical studies (e.g. [7–11]).

Most theoretical studies have identified ecologically relevant mechanisms that explain why the destabilization effect of prey enrichment is seldom observed under field conditions. In general, mechanisms that reduce the per predator consumption rate as predator density increases are thought to weaken the paradox of enrichment [12]. For example, both inducible defensive morphs or predator-avoidance behaviour in prey [13,14] and aggressive mutual interference or ‘prudence’ in predators [15,16] are predicted to be potential resolutions of the paradox in simple predator–prey systems. In more complex communities, including those with multiple prey populations or with spatial structure, theory predicts that imperfection in optimal diet switching by predators (e.g. [17,18]), existence of inedible or invulnerable prey (e.g. [19,20]), diversity in interaction types among species [21], and migration between different patches [22] would aid in community persistence.

However, most previous predator–prey studies have considered an asocial forager that searches for food resources without using socially transmitted information, assuming asocial foraging is simple and mathematically tractable, which could be a valid assumption for some communities, such as phytoplankton–zooplankton systems (e.g. [6,7,20,23]). However, ecological communities often contain many gregarious species, whose foraging behaviour should be influenced by information that comes from conspecifics.

Scrounging (or kleptoparasitism) is a well-known consequence of social information use in predatory species [24,25]. Assume that an individual in a group engages in predation either by searching its environment for food clumps by itself (‘producing’), or visiting other foragers’ food clump discoveries and sequestering some food at each clump (‘scrounging’). Note that scrounging does not require any aggressive interference between predators, and can occur even in aggregations of animals that do not have social structures or genetic relationships [25]. Rather, scrounging exists under any circumstances in which animals search for food, and what information an individual has found or captured is available to conspecifics. For this reason, scrounging should be a common phenomenon in ecological communities and has been documented in many animal species including insects (e.g. [26,27]), fish (e.g. [28,29]), birds (e.g. [30–32]) and mammals (e.g. [33,34]) including humans (e.g. [35–37]).

The game theoretic model of producer–scrounger (PS) behavioural dynamics predicts that both producer and scrounger tactics can stably coexist at an equilibrium [24,25,38–40]. The equilibrium proportion of the two tactics may have a substantial influence on predator–prey population dynamics because prey are discovered only by producing predators. The proportion of producers in the predator population should, therefore, crucially affect predation pressure (i.e. predator–prey encounter rate).

Conversely, population dynamics may affect the equilibrium proportion between the two PS game tactics (i.e. producers and scroungers). In the basic PS game model, an evolutionarily stable state (ESS [41]) of the proportion of producers decreases, and hence that of scroungers increases, with increasing group size [24] (the mathematical expression of this density dependence of producer–scrounger is shown in §(a)). Many empirical studies have supported this group size dependence on the producer–scrounger tactic proportion (e.g. [27,30]). Therefore, the PS game behavioural dynamics and predator–prey population dynamics are likely to interact with each other.

Although both the paradox of enrichment and producer–scrounger dynamics have separately received substantial attention by ecologists, the relationship between them remains unclear. A notable exception is a study by Coolen *et al.* [42], which demonstrates that scrounging behaviour in predators could stabilize the oscillation of the classical Lotka–Volterra predator–prey model. As mentioned above, an increase in predator population density (i.e. group size) reduces the proportion of producers, and a reduction in the producer proportion should reduce the *per capita* predation rate. Therefore, predation pressure is mitigated as the predator population grows, and consequently the Lotka–Volterra system is stabilized [42]. Although the Lotka–Volterra model is too simple to explain complex communities and does not contain the paradox of enrichment because of the absence of prey carrying capacity, Coolen *et al.* [42] suggest that the PS game in predators is a strong candidate for the resolution of the paradox of enrichment in more complex predator–prey systems.

**Figure 1.**
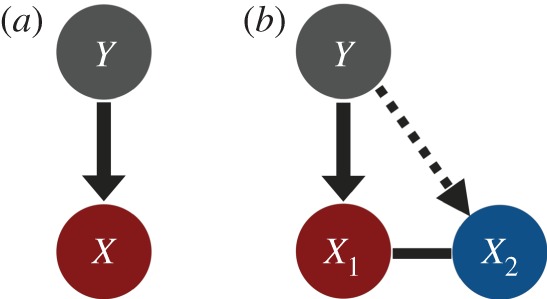
Schematic diagram of the food-web structures of the basal models. (*a*) One-prey-one-predator system (Rosenzweig–MacArthur model [1]); and (*b*) two-prey-one-predator system (Genkai-Kato & Yamamura model [43]). Solid arrows represent fixed links between a predator and a prey species. The dotted arrow represents potentially flexible link between the predator and the less profitable prey-2. The line linking the two prey populations represents the presence of interspecific competition.

In this article, I extend the model of Coolen *et al.* [42] to two different predator–prey systems. First, I used a standard one-prey-one-predator model (i.e. Rosenzweig–MacArthur model [1]; figure 1*a*) and focused on local stability of a coexistence equilibrium point. Classically, resolutions of the paradox have been approached by investigating whether ecologically relevant modifications on the basal predator–prey model could shift the dynamics from limit cycles to a stable equilibrium state (e.g. [13,19,20,44]). Second, I used a two-prey-one-predator system which exhibits non-equilibrium dynamics [43,45–47] (figure 1*b*), and investigated whether the amplitude of population oscillation could be decreased and minimum population density could be increased with increasing enrichment. Studying systems that exhibit non-equilibrium dynamics, rather than studying stability of equilibrium points, is also important for understanding sustainability of complex communities [48–50]. Indeed, many recent studies on the paradox of enrichment have focused on non-equilibrium dynamics [18,43,50–53]. Therefore, the robustness of my conclusion would be increased by investigating both equilibrium and non-equilibrium systems. Herein, I demonstrate that scrounging behaviour in predators may resolve the paradox of enrichment in both systems under a broad range of conditions.

## 2. One-prey-one-predator system

### 2.1. The model

First, I investigated a standard predator–prey model consisting of a prey population *X* and a predator population *Y* [1] (figure 1*a*). I followed the assumptions in Coolen *et al.* [42] to model PS game dynamics in predators as follows. I hypothesized that the predator population was divided into *g* (*g*∈{1,2,3,…}) groups of *G* individuals each (*Y* =*gG*), in which individuals play the PS game with conspecifics. Predator individuals can search for food by either producing (i.e. searching for food asocially and capturing it by themselves) or scrounging (i.e. waiting for other individuals to find food and then stealing it). Each producer (see Glossary) obtains a finder’s advantage *f* out of *F* energetic units (0≤*f*≤*F*) before the scroungers arrive. Once a producer captures a prey item, all of the scroungers arrive and divide the remaining *F*−*f* energetic units equally among the individuals present. Note that producers cannot get any rewards from other producers’ discoveries because they are too busy searching for food to attend to others’ clump (e.g. [32]). I assumed that prey discovery is rare so that no more than a single prey clump is available at one moment (i.e. no more than one prey discovery event can happen at exactly the same time in a time scale of behavioural interaction among foragers); therefore, all scroungers can visit all prey discovery events. Under these assumptions, the evolutionarily or behaviourally stable equilibrium [41] for the proportion of producers among the predators is q*=*g*/*Y* +*f*/*F* (0≤*q*≤1) [24,25]. Notably, because the proportion q* cannot be greater than 1, the model is ecologically relevant when it satisfies 1≤*g*≤*Y* (1−*f*/*F*). I further assumed that behavioural plasticity (e.g. learning) allows individuals to adjust to the PS game equilibrium q* within a time scale much shorter than that of the predator’s birth and death processes [54], such that *q* is always equal to q* in the following one-prey-one-predator dynamics:

2.1a $$\frac{dX}{dt}=\left\{r\left(1-\frac{X}{K}\right)-\mu qY\right\}X$$

and

2.1b $$\frac{dY}{dt}=(b\mu qX-d)Y,$$

where *μ*=*a*/(1+*ahX*) and *q*=q*=*g*/*Y* +*f*/*F*.

*μ* is the instantaneous *per capita* rate of prey capture, depicted in a Holling type II functional response with searching efficiency *a* and handling time *h*; *b* is the conversion efficiency, which relates the predator’s birth rate to prey consumption; and *d* is the death rate of the predator. Note that producing individuals (q*Y), but not scroungers, influence the number of prey captured. For prey population *X*, *r* is the *per capita* intrinsic growth rate, and *K* is the carrying capacity. *K* traditionally indicates the degree of enrichment [2,55,56].

### 2.2. Method

To analyse the effect of scrounging behaviour in predators on the paradox of enrichment, I focused on the proportion of the finder’s advantage *f*/*F* [42]. As seen in the equation q*=*g*/*Y* +*f*/*F*, the finder’s advantage *f*/*F* determines the equilibrium proportion of producers, and hence that of scroungers. The smaller the finder’s advantage, the more prominent the effect of scrounging should be.

I analysed the local stability around a coexisting equilibrium point at which both prey and predator densities are positive. It is well known that local stability can be analysed graphically in the predator–prey phase plane. In the classic Rosenzweig–MacArthur model, the equilibrium is stable if the vertical predator isocline (d*Y*/d*t*=0) crosses to the right of the hump in the prey isocline (d*X*/d*t*=0), whereas it becomes unstable when the predator isocline crosses to the left of the hump [1,57]. Because an increase in *K* does not affect the predator isocline, the increase in *K* will eventually cause the predator isocline to cross to the left of the hump, making the equilibrium unstable (the paradox of enrichment). Herein, I present a similar graphical analysis to show the relationship between equilibrium stability and prey enrichment.

### 2.3. Result

**Figure 2.**
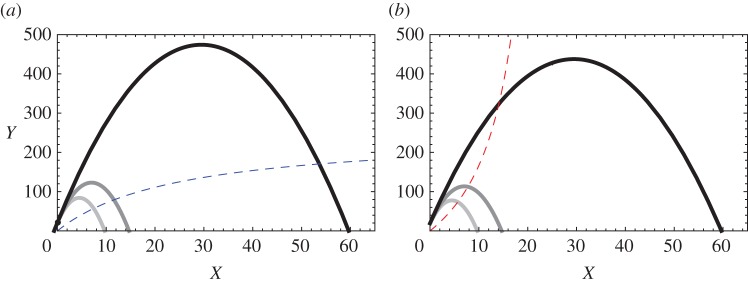
Phase-plane diagrams. Hump shaped solid lines are prey isoclines with different carrying capacities (light grey: *K*=10, grey: *K*=15, black: *K*=60). Dashed lines are predator isoclines, when (*a*) the finder’s advantage is small (*f*/*F*≤*dh*/*b*) and (*b*) the finder’s advantage is large (*f*/*F*>*dh*/*b*). The intersections of the isoclines are coexistence equilibria. The predator isocline is the same for all carrying capacity levels. Parameters were set to the following values: *r*=15, *a*=1, *b*=0.5, *h*=1, *d*=0.25, *F*=1, *g*=5, *f*=0.48 for (*a*) and *f*=0.52 for (*b*).

Figure 2*a*,*b* shows both predator and prey isoclines of the model (equation (2.1)). When the finder’s advantage is sufficiently small (i.e. *f*/*F*<*dh*/*b*; figure 2*a*), the predator isocline is concave-down and never intersects to the left side of the hump in the prey isocline (appendix A); consequently, the predator and prey can stably coexist regardless of prey enrichment. Figure 2*a* also shows that the equilibrium densities of both species increase with enrichment when *f*/*F*<*dh*/*b*. Therefore, the paradox of enrichment disappears if scrounging behaviour is prominent in the predator population.

On the other hand, when the finder’s advantage is large (i.e. *f*/*F*>*dh*/*b*), the predator isocline is concave-up and the intersection point will eventually shift to the left side of the hump as *K* increases. Hence, the paradox still exists (figure 2*b*).

The biological reason why the relative magnitude between the finder’s advantage (*f*/*F*) and *dh*/*b* is crucial in this model can be explained as follows. Suppose the prey density is very high. In that case, the number of prey captured per individual predator is almost maximized to 1/*h* (i.e. $\lim_{X\to \infty }aX/(1+ahX)=1/h$). Given such the circumstance, the predator population dynamics can be written as

2.2 $$\begin{array}{rl} \frac{dY}{dt} & \;=\frac{1}{h}[bq^{*}-dh]Y\\ & \;=\frac{1}{h}\left[b\left(\frac{f}{F}+\frac{g}{Y}\right)-dh\right]Y. \end{array}$$

This clearly shows conditions under which the predator population increases or decreases. Obviously, when the birth rate *b* is smaller than the magnitude of death process *dh*, (bq*−*dh*) is always negative because q*≤1, and the predator population can never persist. When *b*>*dh*, on the other hand, the sign of (bq*−*dh*) depends on the proportion of the producers q*=*f*/*F*+*g*/*Y* . The producer proportion decreases and asymptotes to *f*/*F* with increasing *Y* , and consequently the predator population dynamics asymptotes to the following formula when the predator density and the prey density are very high

2.3 $$\frac{dY}{dt}=\frac{1}{h}\left[b\left(\frac{f}{F}\right)-dh\right]Y.$$

Note that the proportion of producers is minimized here (i.e. q*=*f*/*F*). If the minimum proportion of producers ( *f*/*F*) is insufficient to produce enough food for the entire predator population to overcome the loss by death process (i.e. if *b*( *f*/*F*)<*dh*), the predator population decreases. In other words, the predator population cannot keep growing when predator density is high even if the prey density is very high. When *b*( *f*/*F*)>*dh*, on the other hand, the predator density keeps growing as long as the prey density is sufficiently high. Therefore, the predator population is self-regulated, and the predator–prey system becomes globally stable when *f*/*F*<*dh*/*b* is satisfied.

## 3. Two-prey-one-predator system

### 3.1. The model

My next model is a familiar two-prey-one-predator system which exhibits non-equilibrium dynamics [18,43,45–47] (figure 1*b*). Let there be two prey species. Let one be both the superior competitor and the preferred prey. The predator prefers prey-1 because it is more profitable, i.e. it provides more energy and/or is easier to handle to eat than prey-2. Let the predator be an optimal forager: prey-2 is preyed upon if and only if the population size of prey-1 declines to a certain threshold level [58,59].

To formalize this, I followed a model used by Genkai-Kato & Yamamura [43] as my basal model. Genkai-Kato & Yamamura [43] studied non-equilibrium dynamics of the basal model, and found that the profitability of prey-2 (i.e. less-profitable prey) regulates the amplitude of population oscillations. Nevertheless, the system is universally unstable and the paradox of enrichment remains prominent under a range of conditions. Therefore, this system is a suitable test bed for investigating the resolution of the paradox of enrichment in non-equilibrium dynamics [18,52]. Assuming the producer–scrounger game in the predator population, I investigated the effect of scrounging on the non-equilibrium dynamics of this system.

The two-prey-one-predator system, which consists of a more-profitable prey (prey-1; *X*_1_), less-profitable prey (prey-2; *X*_2_) and predator *Y* , is described as follows:

3.1a $$\frac{dX_{i}}{dt}=\left\{r_{i}\left(1-\alpha_{i1}\frac{X_{1}}{K_{i}}-\alpha_{i2}\frac{X_{2}}{K_{i}}\right)-\mu_{i}qY\right\}X_{i}$$

and

3.1b $$\frac{dY}{dt}=\{b(\varepsilon_{1}\mu_{1}X_{1}+\varepsilon_{2}\mu_{2}X_{2})q-d\}Y,$$

where $\mu_{i}=p_{i}a_{i}/(1+\sum_{j\in \mathrm{preys}}p_{j}h_{j}a_{j}X_{j})$.

For predator population *Y* , *μ*_*i*_ is the instantaneous *per capita* capture rate for prey *i* (*i*∈{1,2}), depicted by a Holling type II functional response; *q* (0≤*q*≤1) is the proportion of producers in the predator population; *a*_*i*_ is the searching efficiency for prey *i*; *h*_*i*_ is the handling time for prey *i*; *ε*_*i*_ is the energy value from an individual of prey *i*; *p*_*i*_ (0≤*p*_*i*_≤1) is the capture probability of an individual of prey *i* given an encounter; *b* is the conversion efficiency, which relates the predator’s birth rate to prey consumption; and *d* is the death rate of the predator species. For prey *i*, *α*_*ij*_ is the intraspecific and interspecific competition coefficients (*α*_*ii*_=1); *r*_*i*_ is the *per capita* intrinsic growth rate of prey *i*; and *K*_*i*_ is the carrying capacity of prey *i*.

#### 3.1.1. Optimal foraging

I assumed that the predator is an optimal forager that maximizes the energy input by predation; in other words, the predator maximizes *ε*_1_*μ*_1_*X*_1_+*ε*_2_*μ*_2_*X*_2_. To do this, the predator can choose the value for the capture probability for each prey *p*_*i*_. As I assume that prey-1 is more profitable, i.e. *ε*_1_/*h*_1_>*ε*_2_/*h*_2_, prey-1 is always included in the diet (i.e. *p*_1_=1) [58]. On the other hand, the capture probability of an individual of the less profitable prey-2 given an encounter, *p*_2_, equals zero or one, depending on whether the density of the more profitable prey-1 is greater or less than the threshold density $\hat{X_{1}}$, where $\hat{X_{1}}=\varepsilon_{2}/\{a_{1}h_{1}h_{2}(\varepsilon_{1}/h_{1}-\varepsilon_{2}/h_{2})\}$ [59]. If the density of prey-1 drops below this critical threshold (i.e. the diet-change threshold), prey-2 is also included in the diet (*p*_2_=1). Otherwise, prey-2 is excluded from the diet (*p*_2_=0). Inclusion or exclusion of the less-profitable prey depends on the difference in profitability (i.e. the expected energy value per handling time *ε*/*h*) and the density of the more-profitable prey.

I further assume that the more profitable prey-1 is superior in competition to the less profitable prey-2 (*α*_12_<*α*_21_), because otherwise the system becomes very fragile (i.e. prey-1 becomes very vulnerable to extinction).

#### 3.1.2. Producer–scrounger game under multiple prey types

Because there are two different prey populations, I had to consider the PS game under multiple food-type scenarios. As in the one-prey-one-predator model described above, I assumed that the predator population (*Y*) is divided into *g* groups, each of *G* individuals (*Y* =*gG*), and the predator individuals can choose either the producing or scrounging tactic. In my two-prey-one-predator system (equation (3.1)), the instantaneous total number of prey captured by a single producing predator is *μ*_1_*X*_1_+*μ*_2_*X*_2_. As in the classic PS game model [24], a producer individual capturing a prey *i* obtains a finder’s advantage *f*_*i*_ out of the maximum *F*_*i*_ energy available before the arrival of scroungers, and then the remaining *F*_*i*_−*f*_*i*_ energetic units are equally divided between the producer and all scroungers present in the group. I also assumed that all scroungers can visit all events of prey discoveries by producers in their group. Expected instantaneous *per capita* energy intakes of both producers (*I*_*p*_) and scroungers (*I*_*s*_) are given by

3.2a $$I_{p}=\sum_{i\in \mathrm{preys}}\mu_{i}X_{i}\left(f_{i}+\frac{F_{i}-f_{i}}{1+(1-q)G}\right)$$

and

3.2b $$I_{s}=qG\sum_{i\in \mathrm{preys}}\mu_{i}X_{i}\left(\frac{F_{i}-f_{i}}{1+(1-q)G}\right),$$

where *q* (0≤*q*≤1) is the proportion of producers.

Setting *I*_p_=*I*_s_ results in a behaviourally (or evolutionarily) stable strategy (ESS; [41]) with producing probability q* (appendix B)

3.3 $$q^{*}=\frac{g}{Y}+\frac{\sum_{i}p_{i}a_{i}X_{i}f_{i}}{\sum_{i}p_{i}a_{i}X_{i}F_{i}}.$$

Note that when *F*=*F*_1_=*F*_2_ and *f*=*f*_1_=*f*_2_, the equilibrium is equal to the that of the original PS game equilibrium q*=*g*/*Y* +*f*/*F* [24,25]. Following the one-prey-one-predator model, I further assumed that behavioural plasticity allows individual predators to adjust to the behavioural ESS within a single time step in population dynamics, such that they always achieve *q*=q* at the time scale of the population dynamics.

### 3.2. Method

Genkai-Kato & Yamamura [43] examined the basal model without a PS game for the predator (figure 1*b*), and showed that the stability of the system was crucially influenced by the profitability of the less profitable prey-2 (i.e. *ε*_2_/*h*_2_). In particular, when the profitability of prey-2 *ε*_2_/*h*_2_ is either very small (‘inedible’) or large (‘palatable’), the system is highly unstable and the paradox of enrichment is prominent. To compare my model with their results, I investigated the non-equilibrium dynamics of my system (equation (3.1)). Because trends in the stability indices were identical for both prey species, I calculated stability for a single species (prey-1 *X*_1_). I focused on the magnitude, along with the minimum density, of population oscillation as indices of the paradox of enrichment. The paradox of enrichment is resolved when amplitudes decrease and minimum densities increase with enrichment [18,53].

### 3.3. Results

**Figure 3.**
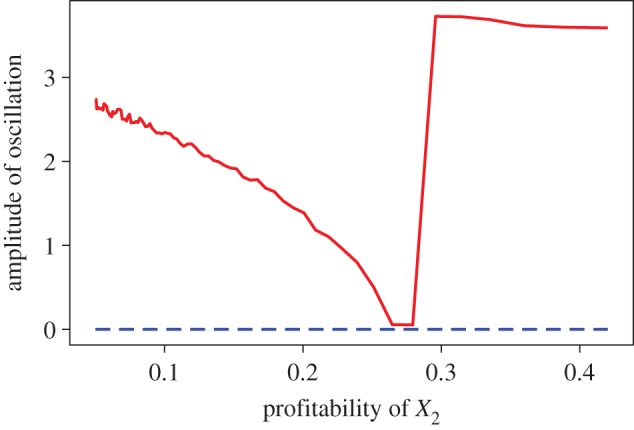
Relationship between the profitability of the less-profitable prey *ε*_2_/*h*_2_ and the amplitude of oscillation defined by the difference between the maximum and the minimum abundance of the more-profitable prey *X*_1_. The dashed line shows the cases in which the finder’s advantage *f*/*F* is either 0.21 or 0.51. The solid line shows the case in which the finder’s advantage *f*/*F* is 0.81. The numerical solution is obtained using the following parameter values: *r*_1_=15, *r*_2_=10, *a*_1_=*a*_2_=1, *ε*_1_=*ε*_2_=0.5, *h*_1_=1, *α*_12_=0.1, *α*_21_=0.4, *b*=1, *d*=0.25, *K*_1_=*K*_2_=4, *F*=1, *g*=3.

Figure 3 shows the magnitude of oscillation relative to the profitability of the less profitable prey-2 *ε*_2_/*h*_2_. For simplicity, here I set *f*=*f*_1_=*f*_2_ and *F*=*F*_1_=*F*_2_. When the finder’s advantage *f*/*F* is small (i.e. *f*/*F*=0.21 or 0.51), the system is always stable regardless of the profitability of prey-2 *ε*_2_/*h*_2_. When the finder’s advantage is large (i.e. *f*/*F*=0.81), however, the system oscillates under a range of conditions as shown in the basal model without the PS game [43]. Note that, when the finder’s advantage is very large (i.e. *f*/*F*≈1), the proportion of the producers is almost always one and the model equation (3.1) becomes identical to the basal Genkai-Kato & Yamamura’s model [43].

**Figure 4.**
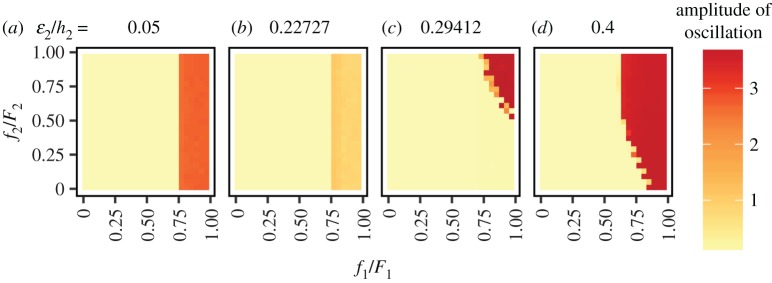
The amplitude of oscillation against the combinations of the finder’s advantages *f*_1_/*F*_1_ and *f*_2_/*F*_2_, at different profitability *ε*_2_/*h*_2_ levels: (*a*) 0.050 (*h*_2_=10), (*b*) 0.227 (*h*_2_=2.2), (*c*) 0.294 (*h*_2_=1.7) and (*d*) 0.400 (*h*_2_=1.25). The oscillation amplitude is shown in different colours from light (yellow) to dark (red). Both *F*_1_ and *F*_2_ were set to 1, and any possible combinations between *f*_1_ and *f*_2_ were tested at step size 0.05. The other parameters are the same as in figure 3.

Next, I considered the differences between the finder’s advantages for two prey species. I examined the magnitude of oscillation against possible combinations of *f*_1_/*F*_1_ and *f*_2_/*F*_2_ for different profitability of the less profitable prey-2 *ε*_2_/*h*_2_. Figure 4 shows that the system is stable under a broad range of combinations of the finder’s advantage. When the profitability of prey-2 *ε*_2_/*h*_2_ is small (figure 4*a*,*b*), the stability of the system relies only on the finder’s advantage for the more profitable prey-1 *f*_1_/*F*_1_. This is because prey-2 has low profitability and is rarely included in the diet. Therefore, *f*_2_/*F*_2_ never affects the predators’ behaviour. On the other hand, when the profitability of the less-profitable prey is large enough, it is included in the predator’s diet, and both *f*_1_/*F*_1_ and *f*_2_/*F*_2_ affect stability (figure 4*c*,*d*).

**Figure 5.**
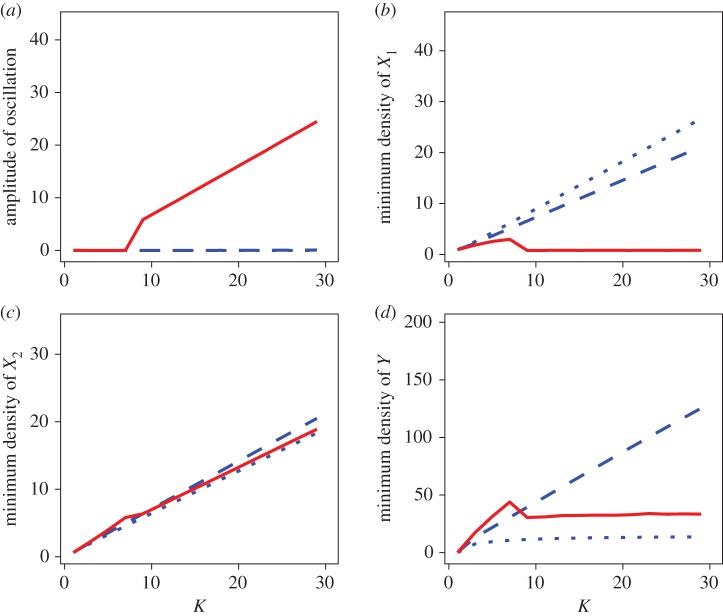
Effect of enrichment with different finder’s advantages (dotted lines: *f*/*F*= 0.3, dashed lines: *f*/*F*=0.5, and solid lines: *f*/*F*=0.6). The degree of enrichment is represented by the magnitude of the prey carrying capacity *K* (=*K*_1_=*K*_2_). (*a*) Relation between enrichment and the amplitude of the oscillation. Note that the dotted line is hidden behind the dashed line. (*b*–*d*) Relation of prey enrichment with (*b*) the minimum density of the more-profitable prey *X*_1_, with (*c*) that of the less-profitable prey *X*_2_, and with (*d*) that of predator *Y* . The same parameter values are used as in figure 3 except for *h*_2_, as *h*_2_=2.2 (i.e. *ε*_2_/*h*_2_=0.227).

Finally, I investigated how the system responds to prey enrichment. For simplicity, I set *K*=*K*_1_=*K*_2_. Figure 5*a* shows that the system remains stable when the finder’s advantage is small (i.e. *f*/*F*=0.3 or 0.5). When the finder’s advantage is large (i.e. *f*/*F*=0.6), however, the system becomes unstable as *K* increases. When the system is stable, minimum densities of all three species increase with an increase in *K* (figure 5*b*–*d*). Therefore, the paradox of enrichment is resolved when the finder’s advantage is small. Interestingly, however, the increase of the minimum density of the predator is weak when *f*/*F* is small compared with when it is at an intermediate-level (see the dotted versus dashed line in figure 5*d*). This is because, when the finder’s advantage *f*/*F* is small, too low a proportion of the predator individuals capture prey (due to the low proportion of the producers), and hence the net energetic gain of the entire predator population is diminished.

When the system oscillates (the solid lines in figure 5), the minimum density of the more profitable prey-1, *X*_1_, nears zero so that stochastic perturbations would lead them to extinction. Therefore, the paradox of enrichment remains when the finder’s advantage is large.

## 4. Discussion

In this study, I demonstrated that social foraging could stabilize both a simple one-prey-one-predator system (figure 1*a*; [1]) and two-prey-one-predator system (figure 1*b*; [43]), and thereby resolve the paradox of enrichment when scrounging behaviour is prevalent in predators. Previous studies have shown that group-living can stabilize an ecological community. For example, group formation in predators may dramatically change functional responses, and hence stabilize predator–prey systems [60]. The ability to monopolize resources by higher-ranked individuals (i.e. social dominance) affects *per capita* food intake rates, which potentially affect population growth [61]. However, few studies have directly addressed a relationship between producer–scrounger foraging dynamics and the paradox of enrichment, although scrounging may be more common than group formation or social dominance in animals.

Using the classic Lotka–Volterra predator–prey model, Coolen *et al.* [42] showed that the system becomes globally stable with the existence of scrounging by predators, without regard to the scroungers’ proportion. On the other hand, my results show that the paradox of enrichment can be resolved only if scrounging is prevalent (i.e. the finder’s advantage *f*/*F* needs to be sufficiently small). As described in the result §(c), the minimum proportion of producers ( *f*/*F*) is crucial for the predator population’s self-regulation (equations (2.2) and (2.3)), and as a result for the system’s global stability. In other words, the proportion of producers changes from *f*/*F* to one, which negatively depends on the predator density *Y* , resulting in the negative density-dependent predator consumption rate.

As Coolen *et al.* [42] discussed, such a stabilizing effect of scrounging by predators is not new. Scrounging is one of the specific mechanisms of predator interference [12]. Predator interference, which refers to any phenomenon in which the *per capita* predator consumption rate decreases as predator density increases, is known to stabilize predator–prey dynamics (e.g. [1,14,15,62]). An intuitive explanation for this stabilizing effect is that a decrease in the individual consumption rate with increasing predator density can prevent the prey population from being overexploited, and hence the population oscillation can be mitigated. Similarly, the core mechanism of the stabilizing effect of the PS game in predators is that the proportion of producers, which contributes to the *per capita* prey capture rate, declines as predator density increases, and hence the overexploitation of prey can be avoided.

In the one-prey-one-predator system, the finder’s advantage *f*/*F* must be smaller than *dh*/*b* to resolve the paradox of enrichment (figure 2). But this inequality is easy to satisfy. Assume that prey handling time *h* becomes longer because of inducible defences or predator avoidance behaviour by prey. A longer handling time may also affect the finder’s advantage because scroungers who have started to approach the captured prey during handling time can arrive as soon as the producer starts to consume the prey. Therefore, factors that lengthen handling time may reduce the finder’s advantage, resulting in an increased chance of stabilizing the system. Conversely, behavioural plasticity in predators may decrease the chance of stability. For example, producers may become more eager to consume prey to compensate losses from kleptoparasitism, which may result in reducing the prey handling time and the chance of satisfying the inequality. Whether (and if so, how) the prey handling time affects the finder’s advantage may depend on the system, which remains open for future empirical studies.

Regarding the two-prey-one-predator system, the profitability of the less profitable prey-2 *ε*_2_/*h*_2_ affects the amplitude of oscillation as shown in the basal model investigated by Genkai-Kato & Yamamura [43] when the finder’s advantage *f*/*F* is large. Conversely, the system can be stable regardless of the profitability of prey-2 *ε*_2_/*h*_2_ when the finder’s advantage is small (figure 3). Interestingly, the finder’s advantage for both prey species contributes asymmetrically to stability (figure 4). The finder’s advantage for the more profitable prey-1 *f*_1_/*F*_1_ influences stability more than that of the less profitable prey-2 *f*_2_/*F*_2_ because of the optimal diet choice by the predator. The more-profitable prey is always included in the diet, whereas consumption of the less-profitable prey is conditional. Therefore, when the profitability of prey-2 *ε*_2_/*h*_2_ is small, the system exhibits similar dynamics to the one-prey model, so that just keeping *f*_1_/*F*_1_ low is sufficient for system stability. When the profitability of prey-2 *ε*_2_/*h*_2_ is intermediate, keeping either *f*_1_/*F*_1_ or *f*_2_/*F*_2_ low is sufficient for stability (figure 4*c*). In this case, the system can be stable even if *f*_1_/*F*_1_ is very high as long as *f*_2_/*F*_2_ is sufficiently low. In summary, my results suggest that scrounging behaviour does not need to be prominent in every predator–prey interaction in the community. Instead, scrounging behaviour that exists only in a subset of the community may be enough to stabilize the food-web as a whole.

I developed the PS game under multiple food-type scenarios (§ii), but they should be tested empirically. Although there have been a large number of empirical studies about PS behavioural dynamics among foragers (e.g. [27–34,40,63]), most of them were conducted using a single type of food resource. The assumption that individuals can reach the ESS in an ecological time scale rests on learning abilities and behavioural plasticity [41,54]. It is probable that animals need greater memory capacity and/or cognitive skills to learn the costs and benefits associated with producing and scrounging when there are different types of foods. Therefore, we need empirical tests to determine whether PS foraging dynamics can emerge in reality under multiple food-type scenarios.

Also, the assumption that the two behavioural tactics are incompatible is crucial for the PS game [32,38,64]. Some empirical results have supported this assumption. For example, nutmeg mannikins (*Lonchura punctulata*) hopping with their head down tend to engage upon food search but not to join others’ discoveries (producing), while those with their head up are looking for other individuals’ discoveries (scrounging) [30]. However, the empirical evidence of the incompatibility assumption is still largely lacking and needs to be tested [64]. Importantly, if individuals conduct food search independently while simultaneously observing one another so as to join others’ discoveries, the other behavioural model, e.g. information-sharing model [65], may be suitable rather than the PS game [38]. To what extent the incompatibility assumption affects my conclusion here is an interesting future direction.

Additionally, the assumption that scrounging is unavoidable might also affect my result. A producer might be able to avoid kleptoparasitism when it is a socially dominant individual [61]. In that case, the discovered resource is monopolized, and as a consequence, the predators distribute as the ideal despotic distribution [66]. Such a modification can alter predator–prey encounter processes, and hence potentially affect the population dynamics.

Although scrounging or kleptoparasitism is well documented in animal aggregations, there are many other phenomena related to social foraging I did not consider. An increasing body of empirical results show that cooperatively sharing information within a group increases foraging efficiency in a colony of social insects (e.g. [67–70]). In addition, opportunities to use inadvertent social information may increase, rather than decrease, the *per capita* food intake rate if the environment is so uncertain that any single individual rarely has very accurate information (i.e. the ‘wisdom of crowds’ effect of information-sharing [71,72]). Enhancing predation efficiency may erase the stabilizing effect of scrounging. Future research will clarify the relationship between this and other aspects of social foraging and stability in a food-web system, and determine which stabilizing or destabilizing effects of social foraging are common in nature.

My study sheds light on the importance of social organisms in community ecology. Recent studies indicate that it is important for community stability to maintain interaction diversity, such as intraspecific and interspecific competition and mutualism, rather than merely protecting the variety of species [21]. My results suggest that it may also be important to consider whether and (if so) how the animals socially interact with conspecifics. Ecologists should be aware of the ubiquitous nature of scrounging behaviour and its potential impacts on community dynamics.

## Supplementary Material

Mathematica code from “Scrounging by foragers can resolve the paradox of enrichment”Description:Mathematica code that can reproduce all the analyses, figures (Fig. 2, 3, 4, and 5), and appendices. The file also contains a replication of the basal two-prey-one-predator model (i.e. Genkai-Kato and Yamamura's model).

## Data accessibility

Mathematica code supporting the results in this study are available as electronic supplementary material.

## Appendix A. Graphical analysis of the stability of one-prey-one-predator model

*X*-coordinate of the coexistence equilibrium is

$$\begin{array}{rl} x^{*} & \;=\frac{-dFr-abfKr+adFhKr}{2((-abfr+adFhr))}\\ & \;\;+\frac{\sqrt{-4(-abfr+adFhr)(adFgK-dFKr)+{\left(dFr+abfKr-adFhKr\right)}^{2}}}{2((-abfr+adFhr))}, \end{array}$$

and the apex of the humped prey isocline is

$$x^{†}=\frac{aKh-1}{2ah}.$$

When *f*/*F*<*dh*/*b*, the coexistence equilibrium point is always on the right side of the hump (i.e. *x**>*x*^†^), and thereby the equilibrium is locally stable. When *f*/*F*>*dh*/*b*, however, *x** is larger than *x*^†^ only if prey carrying capacity is sufficiently small as *K*<( *f*/*F*+*dh*/*b*)/*ah*( *f*/*F*−*dh*/*b*) and when *K*>( *f*/*F*+*dh*/*b*)/*ah*( *f*/*F*−*dh*/*b*) the equilibrium is on the left side of the hump. Therefore, the equilibrium point becomes unstable as *K* increases. Note that whatever *f*/*F*, the coexistence equilibrium never exists unless *r* is sufficiently large as follows:

$$r>\frac{4a^{2}ghK(dh/b)}{\left(\;f/F+2ahK)(dh/b)+a^{2}h^{2}K^{2}(dh/b-f/F\right)}.$$

These results were obtained by using Mathematica software. The code is available as electronic supplementary material.

## Appendix B. Evolutionary stability of *q** in the PS game for multiple prey types

Consider the difference in the expected food intake *D*(*q*)=*I*_p_−*I*_s_. By definition *D*(*q**)=0; and evolutionary, stability requires ∂*D*(*q*=*q**)/∂*q*<0. Differentiating and substituting, I obtain

$${\frac{\partial D(q)}{\partial q}|}_{q=q^{*}}=-\frac{{\left(\sum_{i}p_{i}a_{i}R_{i}F_{i}\right)}^{2}}{\sum_{i}p_{i}a_{i}R_{i}(F_{i}-f_{i})(1+\sum_{i}p_{i}h_{i}a_{i}R_{i})}<0.$$

Hence, *q** is stable as (*F*_*i*_−*f*_*i*_)>0.

## Glossary

– *Producer*. A predator’s behavioural tactic, that is, searching for and capturing a food for themselves and then eating it. Note that in this paper it does not mean an ‘energetic producer’ in a lower trophic level of an ecological community (e.g. plants or phytoplankton).
– *Scrounger*. A predator’s behavioural tactic, that is, never searching for food but rather surveying other individuals and always joining to consume part of each captured prey.
– *Finder’s advantage*. A number of food items (or energetic units) that the producer of a food patch exclusively obtains before the scroungers arrive.
